# Preoperative low dose NSAID treatment influences the genes for stemness, growth, invasion and metastasis in colorectal cancer

**DOI:** 10.3892/ijo.2014.2686

**Published:** 2014-09-30

**Authors:** CHRISTINA LÖNNROTH, MARIANNE ANDERSSON, ANNIKA G. ASTING, SVANTE NORDGREN, KENT LUNDHOLM

**Affiliations:** Department of Surgery, Surgical Metabolic Research Laboratory at Lundberg Laboratory for Cancer Research, Sahlgrenska University Hospital, University of Gothenburg, SE 413 45 Gothenburg, Sweden

**Keywords:** colorectal cancer, clinical trial, NSAIDs, stemness markers, tumor suppressors

## Abstract

Preclinical data, and an increasing list of clinical investigations, show anti-inflammatory agents to favourably influence the biology of colorectal tumor. We have earlier reported on re-expression of activated immune cells after three days preoperative treatment of patients with colorectal carcinoma, randomized to receive oral NSAID (indomethacin or celebrex). Antisecretory prophylaxis (esomeprasol) was provided to all patients and served as sham treatment. Concomittant to MHC locus activation, Prominin1/CD133, a marker associated with stemness and poor prognosis in several solid tumors, was downregulated. The aim of the present study was to evaluate expression of additional regulators belonging to the stem cell niche, OCT4, SOX2 and BMP7, as well as some microRNAs, reported to act as tumor suppressors or oncomiRs. Peroperative tumor biopsies were analyzed by microarrays, quantitative real-time PCR and immunohistochemistry (IHC). The stem cell master regulator SOX2 was increased by NSAIDs (p<0.01), as well as the tumor suppressor miR-630 (p<0.01), while BMP7, a marker for poor prognosis in CRC, was downregulated by NSAID (indomethacin, p<0.02). The upregulation of SOX2, but not of its heterodimer binding partner OCT4, could imply a negative feed-back loop, with a switch-off for stemness preservation of tumor cells. This is supported by the overall evaluation of gene expression profiles with subsequent events, indicating less aggressive tumors following NSAID treatment.

## Introduction

Randomized clinical trials and observational studies have shown NSAIDs to reduce the risk for colorectal cancer and colon adenoma, and also to improve survival in CRC patients, when starting at the time of diagnosis with intake of aspirin/NSAIDs (1–3). Aspirin, provided to stage I-III CRC patients, with or without additional adjuvant chemotherapy, seemed to be associated with improved outcome (4). This is consistent with our own research, indicating reduced tumor progression and improved quality of life following indomethacin treatment (5). Patients with tumors overexpressing COX-2, were reported to have the greatest improvement of survival in one study, while the beneficial effects of aspirin could not be attributed to high expression of COX-2 in another study, where HLA class I antigen appeared a covariate (4,6).

We have also reported that standard oral administration of NSAIDs for three days preoperatively to CRC patients, changed tumor mRNA and protein expression in a biologically favourable direction, when analyzed on whole human oligo microarrays and confirmed by Q-PCR and immunohistochemistry (IHC). Array results, selected for significant up- and downregulation, as viewed in a chromosomal map, exhibited a prominent transcriptional activation at position 6p21, confining the MHC locus. Oral NSAID provision upregulated several genes in this locus followed by increased tumor infiltration of seemingly activated immune cells (7). In further studies based on the same CRC patients, we found mRNA and the AC133 protein epitope expression of Prominin-1/CD133, a marker associated with stemness and poor prognosis in several solid tumors, to be downregulated, including some additional stem cell-related genes, which belonged to the families of WNTs and bone morphogenetic proteins (BMPs) (8–10).

In the present study we have continued analyses on the same patient groups as described earlier, on preoperative NSAID treatment (7,8). We aimed to evaluate mRNA expression of two master regulators of stem cells, the transcription factors OCT4 (POU5F1) and SOX2, as well as BMP7, another gene belonging to the stem cell niche, in an effort to explain decreased levels of PROM1/AC133 during NSAID treatment (11,12). We also aimed to evaluate if NSAIDs would have influence on microRNAs, reported to act as tumor suppressors or oncomiRs in solid tumors (13,14).

## Materials and methods

### Patients

Patients aimed at primary and curative resection of colorectal cancer were randomized to receive NSAID or sham-treatment during three days before surgery, between 1998 and 2004 at Sahlgrenska University Hospital, Gothenburg, Sweden, as reported earlier (7,8). After closure of the study 2004, with the group comprising 14 NSAID-treated patients and 14 sham-treated controls, four patients were further randomized to receive indomethacin or sham-treatment. Two of these patients were included in the present study, while two patients were omitted due to low quality of tumor RNA. The original patient group, now extended by two patients, consisted of 18 females and 12 males with a median age of 73±11 (SD) years (range, 42–85 years). Tumors were histologically classified by certified pathologist as Dukes A (n=5), Dukes B (n=12), Dukes C (n=10) and Dukes D (n=2) corresponding to stage I–IV. One patient had villous adenoma but remained in the study. Tumor stages within groups were: in controls (n=15), Dukes A (n=2), B (n=8), C (n=4), D (n=1) and in NSAID-treated patients (n=15), Dukes A (n=3), B (n=4), C (n=6), D (n=1), villous adenoma (n=1); NSAID treatment was indomethacin (Confortid, 50 mg × 2; Alpharma, n=11) or celebrex (100 mg × 2; Pfizer, n=4) during three preoperative days together with gastric prophylaxis (Nexium 40 mg × 1; AstraZeneca, n=15), which was also provided as sham treatment to all control patients. None of the patients received radiochemotherapy pre- or postoperatively according to our local guidelines or patient preferences (Table I).

### Assessment of medication efficacy

Lymphocyte stimulation was used to confirm the effect of white peripheral blood cells to produce PGE_2_ after endotoxin (LPS) challenge *in vitro* as a surrogate marker of treatment efficacy, following NSAID provision *in vivo*. Peripheral venous blood was drawn from healthy volunteers before and after three days consumption of NSAID as described earlier (7).

### Tumor tissue material

Tumor tissue samples (down to the serosa layer) were collected at surgery, snap-frozen in liquid nitrogen and stored at −70°C until analysis. Recent samples were kept in RNA later (Ambion) for 24 h at 4°C and kept at −20°C until analysis of RNA expression. For IHC, biopsies were kept in 4% buffered formaldehyde solution for three days at 4°C, washed and kept in 70% ethanol until dehydration and paraffin-embedding.

### RNA extraction and cDNA synthesis

Total RNA was extracted with RNeasy Fibrous Tissue Midi kit (Qiagen), where DNase treatment was included according to kit protocol. Quality and quantity of RNA were checked in Agilent 2100 BioAnalyzer with RNA 6000 Nano Assay kit (Agilent Technologies). Concentration of RNA was also measured in a Nano Drop ND-1000A spectrophotometer (Nano Drop Technologies, Inc.). Aliquots of total RNA were used for real-time PCR, where 1 μg total RNA was reverst transcribed with ClonTech First-Strand™ cDNA Synthesis kit (Becton-Dickinson) and incubated for 1 h at 42°C followed by 5 min at 94°C. Each sample was diluted to a final volume of 100 μl. Reactions were run in parallel where the reverse transcriptase was omitted as control for DNA contamination. Poly(A^+^)RNA was selected with mRNA Purification kit (Amersham Biosciences) for microarray analysis. Selected poly(A^+^)mRNA fractions were checked in the BioAnalyzer and quantified in the NanoDrop.

MicroRNA was extracted with mirVana total RNA isolation kit (Ambion/Applied Biosystems) and quality and quantity were checked as described above. cDNA was generated by the miScript II RT kit, where miScript HiSpec Buffer ensured selective conversion of mature miRNAs, used as templates for real-time PCR with the miScript SYBR^®^-Green PCR kit (Qiagen).

Tumor mRNA was pooled from six indomethacin-treated patients and from six sham-treated controls, respectively [indo, 71±11 (SD) years; ctrl, 74±5 (SD) years, two males, four females in each group; Dukes A (n=1), B (n=2), C (n=3) in each group] for microarray analysis.

Tumor miRNA pools were from the same patients, indomethacin and controls, respectively, as described above, minus one in each group due to degradation [indo, 69±10 (SD) years; ctrl, 73±6 (SD) years; celebrex, 72±13 (SD) years]. Tumor miRNA from three celebrex-treated patients was pooled for microarray screening.

### Microarray expression profiling

Pooled mRNA (400 ng) from indomethacin-treated patients were labeled with Cyanine 3-dCTP (Amersham BioSciences) in a cDNA synthesis reaction with Agilent Fluorescent Direct Label kit (G2557A). Four-hundred nanograms of pooled mRNA from control patients were labeled with Cyanine 5-dCTP in parallel to the test-fraction. Expression array (Whole Human Genome Oligo Microarray, G4112A; Agilent Technologies), containing 44,290 features, including positive and negative control spots, was used. Hybridization was performed for 18 h with test versus control cDNA in a dual-color experiment followed by post-hybridization washes according to ‘*in situ* Hybridization kit Plus’ instructions (Agilent Technologies). Microarrays were dried with nitrogen gas in a laminar flow bench and images were quantified on Agilent G2565 AA microarray scanner and fluorescence intensities were extracted using Feature Extraction software program (Agilent Technologies). Dye-normalized, outlier- and background-subtracted values were analyzed in GeneSpring software program, imported with the FE Plug-in (Agilent Technologies). Three technical replicates were run including dye-swap. Informative features from pooled RNA were 41,059 out of 44,290. Hands-on-variation was checked in a ‘yellow experiment’ where the same tumor RNA was labeled with both dyes competing for the same targets.

For miRNA expression profiling, 120 ng of pooled total RNA was labeled with Agilent Cyanine 3-pCp reagent for direct labeling by Agilent microRNA Labeling Reagent and Hybridization kit (Agilent Technologies). Labeled products were hybridized to Agilent Human microRNA (V2) single color microarrays (G4470A; Agilent Technologies), washed and scanned on an Agilent scanner. Analyses of scanned images from single-color microRNA expression were performed in Feature Extraction 9.5 program (Agilent Technologies). Three technical replicates were run for indomethacin and two for celebrex and controls, respectively. The microarray contained 470 human and 64 human viral microRNAs. From the GeneView file, one of the result files from the one channel Feature Extraction program, with background adjusted values and microRNAs passing the QC metrics, some miRNas were chosen for confirmation of expression on a patient individual basis.

### Quantitative real-time PCR

Real-time PCR was performed in a LightCycler 1.5 with either LightCycler FastStart DNA Master (SOX2b, with 2 mM MgCl_2_, final concentration); LightCycler FastStart DNA Master^Plus^ (both from Roche Diagnostics) [PROM1, BMP7, OCT4B, OCT4B1, OCT4B/B1, SOX2a and glyceraldehyde-3-phosphate dehydrogenase (GAPDH)]. Primers for target genes were added to each capillary in a final concentration of 0.5 μM. Primer sequences, fragment length and gene accession number are provided in Table II. For each amplification 2 μl cDNA was used with following PCR conditions: activation for 10 min at 95°C and denaturation for 10 sec at 95°C, 20°C/sec were the same for all mRNAs. Annealing: 7 sec at 58°C (PROM1); 4 sec at 64°C (BMP7, SOX2a, GAPDH); 5 sec at 66°C (OCT4B, OCT4B1, OCT4B/B1); 5 sec at 60°C (SOX2b). Extension and cycle numbers: 22 sec at 72°C, 40 cycles (PROM1); 5 sec at 72°C, 45 cycles (BMP7, SOX2b); 11 sec at 72°C, 45 cycles (OCT4B, OCT4B/B1); 20 sec at 72°C, 40 cycles (OCT4B1); 17 sec at 72°C, 40 cycles (SOX2a); 5 sec at 72°C, 40 cycles (GAPDH).

PCR conditions for miRNAs were as follows: activation for 15 min at 95°C; denaturation for 15 sec at 94°C, 1°/sec; annealing for 30 sec at 55°C; extension for 30 sec at 70°C, 45 cycles.

For mRNA, all samples were performed in duplicates and related to the expression of GAPDH (QuantiTect Primer Assay; Qiagen) which was the least variable housekeeping gene of 11 tested candidates (15). For miRNAs, samples were also performed in duplicates but related to the expression of RNA, U6 small nuclear 2 (RNU6-2). Quantitative results were derived by use of the relative standard curve method where the standard specimen was cDNA from a sham-treated human colon tumor (intermediate differentiation, Dukes C) or pooled cDNA from five tumors from sham-treated patients, belonging to the study. All PCR products had expected size analyzed with Agilents BioAnalyzer in DNA 1000 Chip and all reactions were confirmed by means of both positive and negative controls (one dilution of standard curve cDNA respective water substituted for cDNA).

### IHC

Formalin-fixed and paraffin-embedded tissue sections (4 μm), were deparaffinized and rehydrated according to standard procedure and rinsed twice in 5 mM Tris-buffered saline (TBS), pH 7.8. Sections were microwave-radiated in 0.01 M Citrate Buffer, pH 6.1 (S1700; DakoCytomation) for target retrieval. Sections were mounted with Shandon Coverplates. Non-specific protein binding was initially blocked with TBS containing 5% fat-free dry milk, followed by the procedure described in EnVision Dual Link System-HRP (K4065; DakoCytomation) (CD133) or with a mix of the EnVision kit and BioSite Histo Plus (HRP) Polymer kit (KDB-10003; Nordic BioSite) (SOX2). Monoclonal mouse anti-human CD133/1 (AC133, 130-090-422; Miltenyi Biotec) and rabbit mAB hSOX-2 (D6D9)^xp^ (BioNordica) were used at 15 and 0.2 μg/ml, respectively as final concentrations. Normal mouse IgG_1_ (DakoCytomation, 0931) and normal rabbit IgG (DakoCytomation, X0903) were used as negative controls, incubated in parallel. Diaminobenzidine (DAB), included in the EnVision kit, was used as chromogen. Counterstaining was performed in Mayer’s hematoxylin and mounting was done in Mountex following dehydration (Histolab Products AB).

Observations of protein occurrence and distribution on antibody stained tissue sections were performed in Nikon eclipse E400 microscope and Digital HyperHAD Color Video Camera (Sony) using Easy Image Analysis software (Tekno Optik AB). A semiquantitative scoring system was used, where estimation of protein distribution area in percent was multiplied with intensity scores (range, 0–5; maximum score, 100×5) for evaluation of immunostaining.

### PGE_2_ analysis

Tumor samples were immediately processed as described earlier and PGE_2_ was measured by radioimmunoassay following extraction (16). Tumors from 11/15 patients in each group were analyzed.

### Statistics

Results are presented as mean ± SEM and median values when appropriate. Non-parametric statistics were used in group comparisons (Mann-Whitney U test and CHI2 test). P<0.05 was considered significant and p<0.10 a trend to significance in two-tailed tests.

This study was approved by the Regional Ethics Review Board, University of Gothenburg, Clinical Trials (NCT00473980).

## Results

Results from indomethacin- and celebrex-treated patients were considered as one group of NSAID-treated patients, according to previous reports (7,8).

### PGE_2_

PGE_2_ concentration in NSAID-treated tumors was 5.05±2.04 ng/g tissue (n=11) compaired to 34.42±11.18 ng/g tissue (n=11) in sham-treated controls. NSAID reduced PGE_2_ levels by 85% (p=0.004).

### PROM1/CD133

Prominin1 tumor mRNA expression was re-analyzed in new biopsies from each of the original tumors to confirm our earlier results, including the two new patients. PROM1 expression levels remained significantly lower in patients treated with NSAID compared to control patients (0.45±0.10 vs. 0.79±0.12; p=0.03) (Table III) (8).

It was confirmed earlier that CD133 protein (AC133 epitope) staining appeared in apical plasma membrane of epithelial tumor gland formations and mostly in lumina where shed cells seemed to spread AC133-containing particles (8). Five of 15 NSAID-treated patients (33%) had tumors that stained positive for AC133 compared to tumors in control patients, where 11/15 (73%) (p=0.001) stained positive. The mean rank of staining scores in NSAID-treated tumors was numerically lower compared to scores in control patients, with a trend to significance (49±24 vs. 82±28; p=0.07, median values 0 vs. 60).

### OCT4B/OCT4B1

Levels of transcription factors OCT4B and OCT4B1 mRNA were not significantly changed by NSAID measured by qRT-PCR (Table III). The second primer set for OCT4B/B1, with discriminating capacity between the two transcripts, confirmed the results obtained with the specific primers (2.36±0.84 vs. 0.85±0.29; p=0.43) for NSAID-treated and control patients, respectively. When amplicons were run in Agilent 2100 Bioanalyzer, the gel image with global setting, showed the relationship between the two isoforms, OCT4B (267 bp) and OCT4B1 (492 bp), based on estimated band intensities. Here, two biopsies from each patient were individually analyzed to cover intra-tumoral heterogeneity.

### SOX2

Two array-probes differently located in the mRNA sequence, indicated different signaling from the pooled RNA fractions, although only one transcript is reported for SOX2. At individual Q-PCR follow-up, probes were chosen to cover the two forms, A and B, seen in NCBI AceView. The same increased expression was seen for both amplicons within the study groups and the results were therefore grouped together. Thus, SOX2 transcripts were significantly upregulated by NSAID compared to sham-treatment (2.36±0.52 vs. 0.55±0.14; p=0.002) (Table III).

IHC confirmed SOX2 protein expression in both tumor tissue from study and control patients. Total scores were estimated as 91±52 and 55±29; p=0.75 (median values 40 and 15) for NSAID-treated versus controls. SOX2 protein was found in tumor epithelium in 4/5 tumor sections of NSAID-treated as well as in controls, but differed in cellular localization, where three and one patient expressed nuclear SOX2, respectively (p=0.03).

### BMP7

Tumor BMP7 mRNA was not significantly altered by NSAID, compared to sham-treatment (0.57±0.36 vs. 0.40±0.08; p=0.18), respectively (Table III).

### MicroRNA

Around 240 miRNAs out of the 470 human and 64 human viral miRNAs on the arrays were expressed in tumors from NSAID-treated patients as well as in control patients. Effects by celebrex seemed to be most intense, with high amplitudes and extensive effects on miRNAs. Upregulated expression of the tumor suppressor miR-630 was confirmed with Q-PCR on individual patient basis, with an NSAID ratio of 4.41±1.35 vs. 0.88±0.26 (p=0.002) for controls. Tumor suppressors miR-1 and miR-133a were also evaluated by Q-PCR on an individual patient basis, without significant alterations (2.02±0.86 vs. 0.88±0.26; p=0.41, miR-1) and (3.30±2.29 vs. 1.21±0.71; p=0.48, miR-133a) for NSAID-treated patients versus controls (Table IV).

## Discussion

The stemness associated marker Prominin 1/CD133 was earlier reported to be decreased at mRNA and AC133 protein epitope level in colon cancer tissue by NSAID (8); and now further confirmed by re-analyses of the same but extended patient material. The AC133 antibody (as well as 293C/AC141) recognizes an epitope on the second extracellular loop of the protein and is frequently used to isolate cancer stem cells(CSCs). Expression of AC133 may serve as an independent, significant marker for prognosis and chemoresistance in colorectal cancer, as well as in other solid tumor types (17–19). The complex Prom1 gene contains five promoters and seven splice variants (SVs) and generate distinct protein isoforms. The AC133 antibody does not specifically recognize a glycosylated epitope as previously suggested (20). Nevertheless, CD133 is a highly glycosylated protein with eight putative N-linked glycosylation sites, and differentiation of cells reflects a change in CD133 glycosylation. Neither promoter activity, nor mRNA- or splice variant expression differ between CSCs and differentiated cancer cells (DCCs). CD133 protein expression is unchanged, but the AC133 and 293C/AC141 epitopes are reduced in glycosylation at differentiation (20). This change seems to mask the epitopes for the antibodies, due to different protein folding or by binding to other proteins (20). Changes in the tertiary structure is also supported as reported (21). In our study, however, the diminished detection of AC133 epitope might reflect an event other than differentiation, since mRNA levels were also reduced.

OCT4 (Chr 6p21.3) and SOX2 (Chr 3q26.3-q27), considered to be the top regulators of the pluripotent network during development, as well as in an increasing list of tumors, form a trimeric complex with target DNA (11,12). Principally, the expression of these molecules declines at differentiation of embryonic stem cells (ESCs), while OCT mainly affects proliferation in CSCs (22). There are multiple isoforms of OCT4 with at least three transcripts (OCT4A, OCT4B and OCT4B1) and four protein isoforms (OCT4A, OCT4B-190, OCT4B-265 and OCT4B-164) (23,24). OCT4B1 can be alternatively spliced and translated into all OCT4B proteins, linking OCT4B1 to OCT4B-mediated functions such as stress response (25). Overexpression of OCT4B1 has been related to poor prognosis in cancer in several studies. Expression of OCT4B1 was upregulated in CRC tissues, compared to adjacent non-cancerous tissues, which suggested OCT4B1 to represent a potential biomarker for the initiation, progression and differentiation of CRC (26). Like OCT4B1, SOX2 is reported to act as an oncogene, being upregulated in CRC, and in esophageal squamous cell carcinoma, where it correlated to poor clinical outcome (27,28). SOX2 could also act as a tumor suppressor gene, being frequently downregulated in gastric cancers, some of which appeared due to epigenetic silencing through DNA methylation. Overexpression of SOX2 by transfection of human gastric cell lines induced cell cycle arrest and induced apoptosis (29). Hypermethylation of SOX2 promoter in endometrial carcinogenesis was correlated to short survival of patients as reported (30).

Hence, SOX2 shows conflicting results on expression in different tumor types. Knockdown of SOX2 impaired growth and tumorigenicity in brain tumor cells, but surprisingly, a 3-fold elevation above endogenous levels impaired proliferation. In DAOY medulloblastoma cells, the ectopical elevation of SOX2 reduced cell density and increased the proportion of quiescent cells and gene markers associated with a more differentiated phenotype. Similarly, elevation of SOX2 in prostate and breast tumor cells reduced the number of viable cells (31). OCT4 and SOX2 induce divergent embryonic developmental programmes, thus being not just an on-off control system (32). So, apparently, OCT4 and SOX2 modulate their own transcription by both positive and negative feedback loops in ESCs and CSCs. While SOX2 overexpression in ES cells was shown to mediate a general inhibitory effect on OCT4:SOX2 target genes, this was not observed with OCT4 overexpression, which seemed to inhibit only its own promoter and the Nanog promoter (11,33). Nanog is a partner to OCT4 and SOX2, cooperating in the stem cell niche (34). We observed no influence by indomethacin on tumor mRNA levels of Nanog, when screened on the microarrays, so this gene was not a focus in our study.

In the present study, Q-PCR on individual patient material showed no significant change of OCT4B transcript variants, while SOX2, the heterodimer binding partner to OCT4, was significantly upregulated at mRNA level by NSAIDs. SOX2 protein levels correlated with mRNA expression, as measured in random samples from each patient group by IHC. So, increased levels of SOX2 transcript and protein could imply a negative feedback loop, resulting in reduced number of CD133 expressing cells. This is in accordance to the study by Qiu *et al*, who showed that NSAID targets oncogenic intestinal stem cells (35). Patients with advanced adenomas were divided into two groups, one with subjects taking NSAIDs (not specified) during the preceding year and one group without NSAID intake. Apoptotic TUNEL-positive cells increased by NSAID and could be detected in cells staining positive for OLFM4, an intestinal stem cell marker (35).

Other molecules, closely connected to the stem cell niche, are the BMPs, acting in the BMP signaling pathway. They are extracellularly secreted ligands, belonging to the transforming growth factor β (TGFβ) superfamily. The expression patterns of BMPs are often altered in several tumors and there may be different response to any given BMP, depending on tumor and cell types. BMP7 is reported to be upregulated in breast cancer, malignant melanoma, hepatocellular carcinoma, esophageal squamos cell carcinoma and in CRC, where it is associated with poor prognosis and low overall survival (36–39). In one study of human gastric cancer, BMP7 was downregulated due to promoter methylation, and reduced levels of BMP7 in lung cancer were correlated to positive lymph nodes in another study, where the BMP7 protein was shown to regulate cell motility and progression, with little impact on the growth of tumor cells (40). A selective effect by indomethacin on BMP7 mRNA levels was observed in the present study, with a 3-fold decrease in expression (0.12±0.04 vs. 0.40±0.08, p=0.02) for indomethacin-treated and control patients, respectively, while this effect was lost on the basis of all NSAID-treated patients. This may indicate discrepant effects by specific and unspecific COX inhibitors.

CSCs are often resistant to chemotherapy and other treatments. Partly this is due to deregulated miRNAs, which may function as tumor suppressors or oncomiRs. A retrospective study, screened for a specific tumor miRNA ‘signature’ correlating with pathological complete response (pCR) after neoadjuvant radiochemotherapy of patients with locally advanced rectal cancer. Fourteen miRNAs were differently expressed in the complete responders compared to non-responders. Two miRNAs, miR-622 and miR-630, were upregulated in tumors from patients with good prognosis and were downregulated in poor responders (41). Two other studies, reported induced growth inhibition and apoptosis by miR-630 in a human pancreatic carcinoma cell line, and induced sensitivity of breast cancer cell lines with resistance to HER-targeting drugs (42,43). NSAIDs upregulated miR-630 expression in the present study, with a 5-fold increase. There are 2,418 targets predicted for miR-630 by the miRanda algorithm and NSAID upregulation of miR-630 might thus influence on tumor growth (44). Other miRNAs, acting as tumor suppressors and frequently downregulated in human solid cancers, are miR-1, miR-133a, miR-133b and miR-206 (45–50). miR-1 and miR-133a form clusters on two chromosomes, at 20q13 and 18q11, producing mature miRs with identical sequences, while miR-206 and miR-133b are located at chromosome 6p12 (51). Within the clusters, these miRNAs often cooperate in regulation of oncogene networks as WNT, MAPK and JAK-STAT, with many target genes in common, but they may also function independently. Due to the reported regulatory teamwork of miR-1 and miR-133a and since there were only three informative patients in the celebrex group, we permitted ourselves to sub-group expression values, where celebrex upregulated miR-1/miR-133a 5.7× (4.4× median value) (6.13±3.27 vs. 1.07±0.38; p=0.02) compared to controls. Silencing of miR-1/miR-133a tumor suppressor genes was reported to be caused by promoter methylation, histone deacetylase (HDAC) activity as well as disruption of actin cytoskeleton events, the latter leading to disturbed chromosomal segregation (49). Restoration of miR-1/miR-133a may induce apoptosis and cell cycle arrest, inhibit migration and invasion of cancer cells. Similar effects were shown for miR-133b (48–51).

Microarray analysis in our study, showed tumor suppressor miR-133b to be upregulated selectively 8.8-fold by celebrex. Our microarray results also showed the oncomiR-552 to be downregulated by NSAIDs, compared to sham-treated controls, 2.6- and 5.0-fold for indomethacin and celebrex, respectively, but they were not confirmed on individual patient basis. OncomiR-552 is commonly upregulated in CRC. Overexpression of this miRNA was associated to lymph node and distant metastasis-positive CRCs suggesting that overexpression of miR-552 could imply poor prognosis. When treating HT-29 colon cancer cells with celecoxib, miR-552 was downregulated 2.1-fold compared to control cells (52).

**Figure 1:**
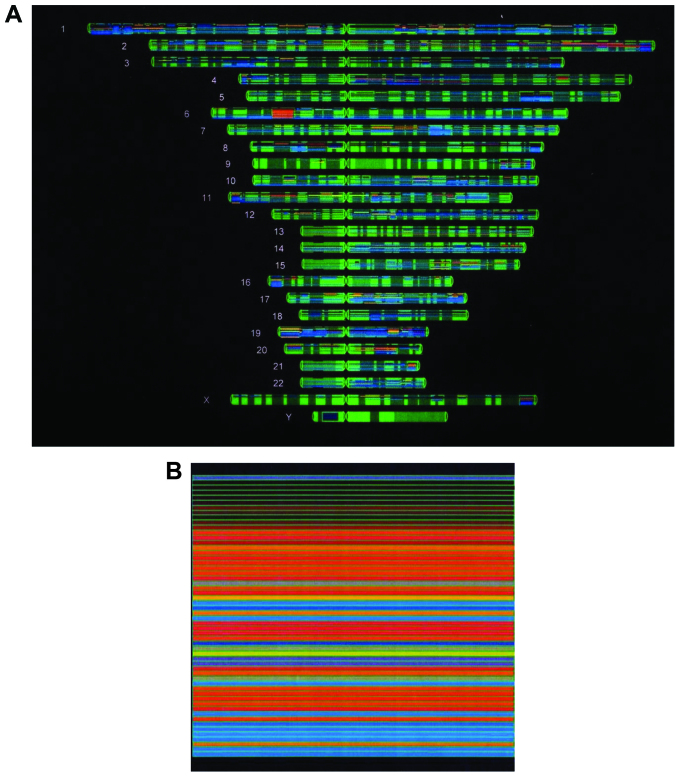
Chromosomal map with (A) physical position view of microarray results with 2-fold up- or 2-fold downregulated genes, in tumors from indomethacin-treated vs. sham-treated control patients, visualized in GeneSpring program (Agilent). (B) Magnification with focus on chromosome 6p21 locus. Upregulated genes are red, downregulated genes are blue.

Due to complex interactions between factors and signaling pathways as judged by visual inspection of files from microarray analyses, we also considered data with indicated different phenotypes of indomethacin-treated patients with support from results in other studies, as well as individually based Q-PCR analyses. Some altered genes belong to chromosome 6p21 or the extended MHC locus (xMHC), covering 7.6 Mb on the short arm of chromosome 6, represented by POLR1C and MAPK14/p38, as well as HIST1H2B1, belonging to a histone gene cluster containing 55 histone genes at 6p22-6p21 (Fig. 1). Together with other genes, they represent tumor markers, growth factors, growth factor receptors, oncogenes, tumor suppressor genes, glucose transporters and cytoskeletal genes (36,53–73) (Table V).

Treatment with a DNA demethylating agent, such as 5-aza-cytidine (5-AzaC), or the histone deacethylation inhibitor trichostatin A (TSA), may induce re-expression of miRNAs followed by expression of protein coding tumor suppressor genes, since deregulated miRNA expression can be caused by epigenetic silencing due to DNA methylation of promoter CpG islands or hypoacetylation of nucleosomal histone proteins (49,51). Several DNA methyltransferases catalyze DNA methylation, e.g., DNMT1, DNMT3a and DNMT3b (74). DNMT1 controls precise duplication and maintains the pre-existing global DNA methylation patterns after duplication in addition to gene-specific methylation in human cancer cells and DNMT3a/b are involved in *de novo* methylation (75,76). Peng *et al* showed DNMT1 protein expression to increase significantly and progressively in multistage carcinogenesis of the pancreas. They analyzed five cell cycle control genes and found tumor suppressor genes APC and CDKN2A/p16 to be the most frequently methylated in their study (77). Selective depletion of DNMT1 was reported to reactivate expression of CDKN2A/p16 in HCT116 colon cancer cells and re-expression of CDKN2A/p16 after knockdown of DNMT1 in human lung and breast cancer cells (74,76,78). Moreover, p16^Ink4^ (CDKN2A) methylation in CRC patients seemed to define a group with poor prognosis (79).

Our microarray data showed that indomethacin appeared to decrease DNMT1, which seemed to be coordinated with upregulation of CDKN2A/p16 tumor suppressor gene (Table V). Therefore, we conclude that upregulation of HLA and accessory molecules, as reported earlier, in part could be explained by the decrease of DNA methylating enzymes.

Pair of four core histone proteins, H2A, H2B, H3 and H4, make up the octameric nucleosomes, around which the DNA is wrapped, with histone 1 as a linker. Histone modifications, the ‘histone code’, affect chromatin structure and gene expression. Histone acetyltransferases (HATs) acetylate the N-terminal histone tail, make a ‘relaxed’ chromatin structure that allows transcriptional activation, and HDACs make the chromatin condensed and inactive for DNA transcription (80,81). In our study, no change in mRNA expression of HATs or HDACs was seen. We found a direct histone-associated event, the downregulation of the H2A histone family, member B3 (H2AFB3) and histone H2B type1-N (HIST1H2BN) at mRNA levels on microarrays (Table V). Histone synthesis is tightly coupled to chromosomal replication during S-phase of cell division (73). H2AFB3 is an atypical histone and can replace conventional H2A in some nucleosomes, making them less rigid, and being associated with active transcription and RNA processing (GeneCards). Inhibition of histone synthesis during S-phase in mammalian cells destabilizes chromatin organization and may induce DNA damage (73).

The non-histone modifier HDAC6 associates with CD133, being the only candidate interaction partner for CD133 (54). CD133, HDAC6 and β-catenin can associate to a complex and might be directly involved in promoting WNT/β-catenin signaling. Depletion of CD133 or HDAC6 in OVCAR-8 cells resulted in reduction of mesenchymal-and metastasis-associated genes such as SLUG, laminin γ1 (LAMC1) and matrix metalloproteinase 7 (MMP-7), reported as targets for the WNT/β-catenin pathway (54). In our present study, the downregulated level of Prom1/AC133 was followed by a decrease of LAMC1 and MMP-7 (Table V). Further, HDAC6 has also been shown to be required for efficient oncogenic RAS-associated transformation and tumor formation (82). CD133 was shown to have similar effects and suggested a possible RAS influenced signaling module of HDAC6 and CD133, for the cell cycle (54,82).

Indomethacin and other amphiphilic NSAIDs stabilize cholesterol domains in the plasma membrane, thereby influencing on membrane heterogeneity and protein nanoclustering, with consequences for cell signaling (56). Ras proteins are anchored to the inner surface of the plasma membrane, where H-, K-, and N-Ras proteins assemble into spatially distinct dynamic nanoclusters. Indomethacin (and other NSAIDs) compromise the GTP-dependent lateral segregation and disturb nano-cluster separation, thereby decreasing Ras signal transmission through the MAPK pathway. Different NSAIDs showed that this membrane stabilizing effect was independent of COX activity (56).

Our microarray results indicated that H-Ras was downregulated 7-fold by indomethacin, whereas K-Ras and N-Ras were unchanged (Table V). Kim *et al* showed that H-Ras induced an invasive phenotype in human breast epithelial cells, by signaling through MAPK/p38 (MAPK14). The increased cell motility was accompanied with ECM degradation by increased activity of matrix metalloproteinases (57). MAPK14 and MMP-7 were downregulated on microarrays by indomethacin in our present study.

The course of events by NSAID treatment is hard to predict, as shown in studies with celecoxib and anti-inflammatory plant compounds as curcumin and quercetin (61,83,84). *In vitro* experiments with only one type of cells, factors as drug concentration and time course, influence on expression and direction. Considering the complex network in colon tumors, with communication between cancer cells and surrounding stromal cells, there are still some genes or group of genes that are frequently occurring as targets for these anti-cancer substances, e.g., POLR1C (transcription regulation), CDKN2A and cyclins (cell cycle), MHC class II genes (immune system), MAPKs, PRKC, BMPs and FGFR (signal transduction), integrins (cell adhesion), CEACAM 5 (oncogene) and histones (cell cycle control). This is in agreement with our overall findings and with the NSAID model network (85) (Table V). Several of these genes belong to the xMHC on chromosome 6p22.2-6p21.32, one of the most polymorphic and gene-dense regions in the human genome. This was recently confirmed in genome-wide association studies (GWAS), where the genome was partioned into 200 kb ‘bins’ in a meta-analysis in an effort to map disease loci. While 92% of bins were not disease-associated, 10 bins (0.06%) were significantly enriched for susceptibility of multiple diseases. Two with highly significant ‘hotspots’ mapped to the MHC locus, 6p21 (four bins), and to the CDKN2a/b (INK4/ARF) tumor suppressor locus on chromosome 9p21.3 (one bin). Surprisingly, 30% of all tested human diseases mapped to one of these two regions. The 10 significantly enriched bins contained genes associated to inflammation or cellular scenescence pathways, including cancer (86).

In our study, tumor PGE_2_-concentrations were depressed by NSAIDs to 15% of controls, mostly due to inactivation of COX enzymes, since no significant changes in mRNA expression of these molecules could be detected. Thus, PGE_2_ downstream signaling through EP-receptors should have been reduced by 85%, implying a more favourable situation for patients, consistent with other reports (1–5).

Also, in spite of the NSAID-upregulated mRNA expression of the pro-pluripotency gene SOX2, other putative stem cell associated markers found in CRC were not increased (87,88). Instead, drug-induction suggested decrease for ITGB1/CD29c and ALDH1B1, while CD24, CD44, CD166, ALDH1A1 and Lgr5 were unchanged as detected by signals from microarrays with pooled tumor RNA from indomethacin-treated patients versus pooled tumor RNA from control patients. Overexpression of these markers correlates to poor prognosis (Table V).

Taken together, a short preoperative NSAID treatment of CRC-patients seemed to decrease expression of several genes responsible for growth, invasion and metastasis and to increase expression of tumor suppressors as well as to activate the immune system. This change towards less aggressive tumor cells may be associated with improved outcome in patients, as reported by us earlier (5). Thus, a growing list of evidence supports the use of anti-inflammatory agents as adjuvant therapy for colorectal cancer patients, improving both survival and quality of life (89).

## Figures and Tables

**Table I:** Patient characteristics before operation in patients randomized to NSAID or sham treatment.

	Patients	
	
	NSAID-treated	Controls
Male/female	8/7	4/11
Age	70±3	75±2
Dukes^a^		
A	3	2
B	4	8
C	6	4
D	1	1
Villous adenoma	1	-
Weight	74±3 (n=13)	75±4 (n=11)
Hb	121±5 (n=12)	113±5 (n=13)
Bi/S	9±1 (n=11)	8±1 (n=11)
ASAT	0.58±0.15 (n=10)	0.43±0.04 (n=11)
ALAT	0.47±0.08 (n=11)	0.31±0.04 (n=11)
S-creatinin	112±7 (n=12)	97±4 (n=12)
Alive, survival, years	10.0±1 (n=8)	9.6±1.4 (n=4)
Tumor differentiation		
Low	5	1
Intermediary	7	11
High	3	1
Tumor location		
Right	8	8
Transverse	3	0
Left, sigmoideum	3	7
Rectum	1	-

Mean ± SEM.

a Corresponding to tumor stages I–IV.

Hb, hemoglobin; Bi/S, serum-bilirubin; ASAT, aspartate aminotransferase; ALAT, alanine aminotransferase.

**Table II:** Gene names and accession numbers, primer sequences, fragment length and suppliers, used in qRT-PCR.

Gene name	Accession no.	Primer sequences	Fragment length (bp)	Supplier
Hs_OCT4B, isoform 2	NM_203289	OCT4B-F(OCT-FB)^a^ 5′-AGA CTA TTC CTT GGG GCC ACA C-3′	244	
		OCT4B-R(OCT-RB5)^a^ 5′-GGC TGA ATA CCT TCC CAA ATA GA-3′		CyberGene
Hs_OCT4B1, isoform 3	EU518650	OCT4B-F(OCT-FB)^a^ same as above	272	
		OCT4B1-R(OCT-RB4)^a^ 5′-CTT AGA GGG GAG ATG CGG TCA-3′		CyberGene
Hs_OCT4B/B1, discr. isoform 2 and 3		OCT4B-F (OCT-FB)^a^ same as above	267 resp.	
		OCT4B/B1 (OCT-RB2)^a^ 5′-CTC AAA GCG GCA GAT GGT CG-3′	492	CyberGene
Hs_SOX2a	NM_003106	SOX2a-F 5′-AAG TTC TAG TGG TAC GGT AGG A-3′	447	CyberGene
		SOX2a-R 5′-ATT ACC AAC GGT GTC AAC CTG C-3′		
Hs_SOX2b	NM_003106	RT^2^ qPCR Primer Assay for human SOX2, PPH02471 A	115	SABiosciensis
		Reference position: base 1091		Qiagen
Hs_BMP7	NM_001719	QuantiTect Primer Assay for human BMP7, QT00068936	128	Qiagen
Hs_Prom1	NM-006017	PROM1-F 5′-TGG ATG CAG AAC TTG ACA ACG T-3′	552	CyberGene
		PROM1-R 5′-TGC TCG TGT AAG GTT CAC AGA T-3′		
Hs_GAPDH	NM_002046	Hs_GAPDH_1_SG QuantiTect Primer Assay, QT00079247	95	Qiagen
Hs_miR-1	MIMAT0000416	miScript Primer Assay for Human miR-1_2, MS00008358	85–87	Qiagen
Hs_miR-133a	MIMAT0000427	miScript Primer Assay for Human miR-133a_2, MS00031423	85–87	Qiagen
Hs_miR-630	MIMAT0003299	QUNTHSMIR-0630	85–87	Quanta BioSciences
Hs_RNU6-2	Entrez Gene ID: 26826	miScript Primer Assay for Human RNU6-2_11, MS00033740	85–87	Qiagen

a Annotation between brackets (24).

**Table III:** Tumor transcript alterations of stem cell-related genes in colorectal cancer from patients randomized to preoperative NSAID treatment versus sham treatment.

Q-PCR ratio	Sham-treated controls	NSAID-treated	P-value^a^
SOX2a	0.20 (n=14)	1.15 (n=14)	**0.03**
	0.59±0.26	2.07±0.56	
SOX2b	0.41 (n=15)	1.18 (n=15)	**0.03**
	0.53±0.14	2.64±0.88	
SOX2a+b	0.27 (n=15)	1.18 (n=15)	**0.002**
	0.55±0.14	2.36±0.52	
OCT4B	1.26 (n=14)	1.17 (n=14)	0.46
	1.77±0.33	1.84±0.51	
OCT4B1	0.08 (n=14)	0.80 (n=14)	0.14
	0.41±0.13	1.17±0.35	
OCT4B/B1	0.24 (n=15)	0.86 (n=15)	0.43
	0.85±0.29	2.36±0.84	
BMP7	0.42 (n=15)	0.22 (n=15)	0.18
	0.40±0.08	0.57±0.36	
PROM1	0.64 (n=15)	0.29 (n=15)	**0.03**
	0.79±0.12	0.45±0.10	

Median values and mean ± SEM.

a Mann-Whitney U test.

**Table IV:** Tumor microRNA alterations in colorectal cancer from patients randomized to preoperative NSAID treatment versus sham-treatment.

Q-PCR ratio	Sham-treated controls	NSAID-treated	P-value^a^
Hsa miR-630/RNU6	0.67 (n=6)	2.44 (n=9)	**0.002**
	0.88±0.26	4.41±1.35	
Hsa miR-1/RNU6	0.57 (n=6)	1.22 (n=9)	0.41
	0.93±0.32	2.02±0.86	
Hsa miR-133a/RNU6	0.57 (n=6)	0.86 (n=9)	0.48
	1.21±0.73	3.30±2.29	

Median values and mean ± SEM.

a Mann-Whitney U test.

**Table V:** Microarray data and tumor gene expression profiles.

A, Gene transcript alterations in microarray analyses of pooled CRC tumors from patients randomized to preoperative NSAID treatment versus sham treatment.				

Systematic name	Gene name	Descriptive name	Array ratio	No. of arrays with p<0.05
NM_006017	PROM1	Hs prominin 1	0.57±0.13	2/3
NM_002293	LAMC1	Hs laminin γ1 (formerly LAMB2)	0.45±0.07	2/3
NM_002423	MMP7	Hs matrix metalloproteinase 7	0.41±0.02	3/3
NM_005343	HRAS	Hs v-Ha-RAS Harvey rat sarcoma viral oncogene homolog	0.14±0.04	3/3
NM_002747	MAPK4	Hs mitogen-activated protein kinase 4	0.28±0.09	3/3
NM_001315 Z25432	MAPK14	Hs mitogen-activated protein kinase 14, transcript variant 1	0.36±0.09	3/3
NM_006129	BMP1	Hs bone morphogenetic protein 1, transcript variant BMP1–3	0.25±0.04	3/3
NM_001718	BMP6	Hs bone morphogenetic protein 6	0.58±0.28	2/3
NM_001719	BMP7	Hs bone morphogenetic protein 7	0.50±0.03	3/3
NM_002737	PRKCA	Hs protein kinase C, α	0.26±0.04	3/3
NM_173500	TTBK	Hs τ tubulin kinase 2	0.19±0.05	3/3
NM_000343	SLC5A1	Hs solute carrier family 5 (sodium/glucose co-transporter), member 1	0.64±0.15	2/3
NM_001379	DNMT1	Hs DNA (cytosine-5)-methyl-transferase 1	0.33±0.15	2/3
NM_058197	CDKN2A/p16	Hs cyclin-dependent kinase inhibitor 2A, transcript variant 3	2.41±0.27	2/3
NM_001759	CCND2	Hs cyclin D2	0.55±0.03	3/3
NM_023110	FGFR1	Hs fibroblast growth factor receptor 1, transcript variant 1	0.22±0.07	3/3
NM_145040	PRKCDBP	Hs protein kinase C, δ binding protein	4.14±1.62	2/3
NM_004935	CDK5	Hs cyclin-dependent kinase 5	0.19±0.06	3/3
NM_004875	POLR1C	Hs polymerase (RNA) I, (DNA directed), polypeptid C, 30 kDa	0.14±0.06	3/3
NM_000149	FUT3	Hs fucosyltransferase 3	0.43±0.08	3/3
NM_004363	CEACAM5	Hs carcinoembryonic antigen-related cell adhesion molecule 5	0.53±0.05	2/3
NM_002483	CEACAM6	Hs carcinoembryonic antigen-related cell adhesion molecule 6	0.41±0.02	3/3
NM_006890	CEACAM7	Hs carcinoembryonic antigen-related cell adhesion molecule 7	0.50±0.12	2/3
NM_000692	ALDH1B1	Hs aldehyde dehydrogenase 1, family member B1	0.36±0.04	3/3
NM_012098	ANGPTL2	Hs angiopoietin-like protein 2	0.15±0.05	3/3
NM_033667	ITGB1/CD29	Hs integrin β1, isoform 1C-1	0.10±0.03	3/3
NM_003520	HIST1H2BN	Hs histone 1, H2 bn	0.23±0.02	3/3
NM_080720	H2AFB3	Hs histone H2A variant Barr-body deficient	0.17±0.07	3/3

B, Deduced tumor gene expression profile with subsequent events following preoperative indomethacin treatment.	

Gene name	Subsequent events
INDO, PROM1, LAMC1, MMP7	Depressed signal transduction with a reduction in expression of mesenchymal-and metastasis-associated genes, which also might be involved in Wnt/β-catenin signaling (54,55).
INDO, H-RAS, MAPKs, MMPs	Depressed signal transduction with decreased motility and invasiveness (56,57).
INDO, BMPs, BMPR1/2^a^, MAPKs	Decreased signal transduction with depressed aggressiveness, less metastases and improved prognosis (36,58).
INDO, PRKCA, TTBK, SLC5A1	Decreased transport, with downregulated protein kinase C and T-tubulin kinase, which regulate the Na^+^-coupled glucose transporter SLC5A1 (SGLT1), able to work at low glucose concentrations. This results in less glucose uptake in tumor cells, influencing their survival (59).
INDO, DNMT1, CDKN2A/P16, CyclinD2	Downregulation of methylating protein DNMT1 reactivates expression of tumor suppressor CDKN2A/P16, which inhibits Cyclin D-CDK complexes, followed by decreased cell proliferation. Less expression of CCND2 at the invasive margins of CRCs probably means lowered risk for metastases (60,61).
INDO, FGFR1	FGFR1 signal transduction is involved in several pathways, influencing angiogenesis, proliferation and cell growth. Downregulation of FGFR1 in tumors means attenuated growth and less metastases (62).
INDO, PRKCDBP	Protein Kinase C, δ binding protein, is a proapoptotic tumor suppressor, known to be frequently downregulated by promoter hyper methylation in CRC. Upregulation induces G(1) cell cycle arrest (63).
INDO, CDK5	Cyclin-dependent kinase 5 is reported to be overexpressed in pancreatic cancer, colon tumors as well as in colon cancer cells, while normal colonic mucosa has minimal expression. Downregulation of CDK5 means decreased cell proliferation (64,65).
INDO, POLRC1	In transcription regulation, polymerase (RNA) I polypeptide C, 30 kDa (POLR1C) provides instruction for making one subunit in RNA polymerase I and III, which are involved in synthesizing rRNA and tRNA. Shortage of rRNA might trigger apoptosis (61).
INDO, CEACAMs	Carcinoembryonic antigen cell adhesion molecules CEACAM5 (CEA) and 6 are often overexpressed in CRC. Downregulation of these tumor markers indicates less invasiveness and metastases. CEACAM7 is reported to have divergent expression in different tumor types (66–68).
INDO, FUT3	The tumor marker galactoside 3(4)-L-fucosyltransferase (FUT3/CA19-9) is often upregulated in CRC and involved in cancer cell adhesion to endothelial cells, as well as in TGFB-mediated EMT. Downregulation decreases tumor cell migration, invasion and the metastatic activity (69).
INDO, ALDH1B1	Aldehyde dehydrogenase 1 (ALDH1B1) is an enzyme and common marker for stem cells and cancer stem cells, responsible for the oxidation of intracellular aldehydes. Downregulation of ALDH1 suggests improved prognosis for CRC patients (70).
INDO, ANGPTL2, ITGB1/CD29c	Angiopoietin-like protein 2 is reported to increase inflammatory carcinogenesis in several cancers, mediated by the integrin receptor α5β1/CD29, with signaling molecules, e.g., MAPK14/p38 and MMPs. Downregulation of these genes means less inflammation, less cell migration and metastases (71,72).
INDO, HIST1H2BN	Histone synthesis is tightly coupled to chromosomal replication during S-phase of cell division cycle, and downregulation of histones might destabilize chromatin organization and induce DNA damage (73).
INDO, H2AFB3	This is an atypical histone, which can replace conventional H2A in some nucleosomes, making them less rigid and being associated with active transcription and RNA processing (GeneCards).

Array ratio mean ± SEM.

a BMPR1/2 transcripts: not changed.
